# Evaluation of serum high‐mobility group box 1 concentration in dogs with epilepsy: A case‐control study

**DOI:** 10.1111/jvim.15940

**Published:** 2020-11-05

**Authors:** Yoonhoi Koo, Hakhyun Kim, Taesik Yun, Dong‐In Jung, Ji‐Houn Kang, Dongwoo Chang, Ki‐Jeong Na, Mhan‐Pyo Yang, Byeong‐Teck Kang

**Affiliations:** ^1^ Laboratory of Veterinary Internal Medicine College of Veterinary Medicine, Chungbuk National University Cheongju Chungbuk Republic of Korea; ^2^ Institute of Animal Medicine, College of Veterinary Medicine, Gyeongsang National University Jinju Gyeongnam Republic of Korea; ^3^ Veterinary Teaching Hospital, College of Veterinary Medicine, Chungbuk National University Cheongju Chungbuk Republic of Korea

**Keywords:** biomarker, canine, HMGB1, seizure

## Abstract

**Background:**

High‐mobility group box 1 (HMGB1) is a key mediator of neuroinflammation and there are increased HMGB1 levels in laboratory animal models of epilepsy and human patients with epilepsy.

**Objectives:**

To determine serum HMGB1 levels in dogs with epilepsy.

**Animals:**

Twenty‐eight epileptic dogs, 12 dogs with nonepileptic brain diseases, and 26 healthy dogs.

**Methods:**

In this case‐control study, serum HMGB1 concentrations were estimated using the canine‐specific enzyme‐linked immunosorbent assay kit. Diagnosis of dogs with epilepsy was based on medical history, physical and neurological examination findings, laboratory test results, magnetic resonance image, and cerebrospinal fluid analysis.

**Results:**

Serum HMGB1 levels were significantly higher in epileptic dogs (median = 0.41 ng/mL; range, 0.03‐5.28) than in healthy dogs (median = 0.12 ng/mL; range, 0.02‐1.45; *P* = .002). In contrast, serum HMGB1 levels of dogs with non‐epileptic brain diseases (median = 0.19 ng/mL; range, 0.03‐1.04) were not significantly increased compared to those of healthy dogs (*P* = .12). Regarding idiopathic epilepsy, dogs with an epilepsy course of >3 months showed a higher serum HMGB1 concentration (median = 0.87 ng/mL; range, 0.42‐2.88) than those with that of ≤3 months (median = 0.26 ng/mL; range, 0.03‐0.88; *P* = .02).

**Conclusions and Clinical Importance:**

Serum HMGB1 could be a biomarker of epilepsy.

**Abbreviations**
BBB: blood brain barrier
BT: brain tumor
CCL: c‐c motif chemokine ligand
CSF: cerebrospinal fluid
FLAIR: fluid‐attenuated inversion recovery
HMGB1: high‐mobility group box 1
IE: idiopathic epilepsy
IL: interleukin
KBr: potassium bromide
MRI: magnetic resonance image
MUO: meningoencephalitis of unknown origin
ROC: receiver operating characteristic
SE: structural epilepsy
TLR: toll‐like receptor
TNF‐α: tumor necrosis factor‐α

## 1 INTRODUCTION

Proinflammatory events lead to seizures in animal models of epilepsy.^1^ Experimental and clinical evidence supports that inflammatory mediators play a key role in epileptogenesis.^2^, ^3^, ^4^ Although currently, there is no consensus on the definition of neuroinflammation, it is commonly recognized as the biosynthesis and release of inflammatory mediators by various brain cells including neurons, activated microglia, and astrocytes, macrophages, and endothelial cells of the blood brain barrier (BBB).^5^ Neuroinflammation induces individual seizures along with cell death, which in turn promotes further development of seizures by activation of inflammatory pathways.^6^ Although neuroinflammation affects glial and neurovascular function, the effects of systemic inflammation are facilitated or mediated by the loss of BBB function.^7^ There are increased levels of several inflammatory mediators including interleukin (IL)‐1α, c‐c motif chemokine ligand (CCL) 4, CCL11, CXCL10, CX3CL1, high‐mobility group box 1 (HMGB1), and basic fibroblast growth factor are associated with neuroinflammation in the serum of human patients with epilepsy.^8^, ^9^, ^10^, ^11^

In veterinary medicine, there are increased IL‐1β and tumor necrosis factor‐α (TNF‐α) levels in serum and IL‐6 levels in the cerebrospinal fluid (CSF) of epileptic dogs,^12^, ^13^ implying that neuroinflammation can be involved in epileptogenesis in dogs.

High‐mobility group box 1 is a ubiquitous nuclear protein related to inflammation, which is released by glial cells and neurons and activated by inflammasome activation.^4^ The release of HMGB1 from glial cells and neurons reduces seizure threshold via HMGB1‐mediated IL‐1β and neuronal toll‐like receptor (TLR) 4 activation in rodent models of epilepsy.^5^ High‐mobility group box 1 expression is reportedly upregulated in the brain tissue of epileptic dogs.^14^

Serum HMGB1 level is potentially involved in the induction and development of epilepsy or epileptic lesions and could be a potential predictive marker for epilepsy prognosis in humans.^15^, ^16^, ^17^ Furthermore, an elevation of serum HMGB1 level is associated with an increase in seizure duration, seizure frequency, and antiepileptic drugs resistance in human epileptic patients.^18^

There are several reports on the correlation between serum HMGB1 levels and certain diseases in dogs, such as lymphoma, systemic inflammatory response syndrome, gastric dilatation and volvulus, and pancreatitis^19^, ^20^, ^21^, ^22^, ^23^; however, among the cerebral diseases of dog, encephalitis is the only disease that correlated with HMGB1 levels in CSF.^24^

This study aimed to establish preliminary support for the use of serum HMGB1 concentration as a potential biomarker for epilepsy in dogs. We hypothesized that the serum HMGB1 concentration would be elevated in dogs with epilepsy, regardless of the etiology. Therefore, this study aimed to elucidate the factors that could affect serum HMGB1 levels including etiology, status, and treatment of epilepsy.

## 2 MATERIALS AND METHODS

### 2.1 Animals

The dogs that visited the Chungbuk National University Veterinary Teaching Hospital between 2015 and 2019 were recruited in this case‐control study. Dogs were determined to be clinically healthy if physical examination, complete blood count (CBC), serum biochemical analysis, serum electrolyte analysis, urinalysis, survey radiography, and abdominal ultrasonography revealed no remarkable findings. These clinically healthy dogs were included in the control group. Dogs presented with signs of cerebral disease underwent physical and neurological examination, CBC, serum biochemical analysis, serum electrolyte analysis, magnetic resonance imaging (MRI), and CSF analysis.

Diagnosis of these dogs was based on characteristics presented in medical records, including the signalment, medical history, physical and neurological examination findings, laboratory test results, and MRI findings. Dogs diagnosed with idiopathic epilepsy (IE), meningoencephalitis of unknown origin (MUO), and brain tumor (BT) were included in this study.

Idiopathic epilepsy was defined as recurrent seizures of unknown origin accompanied by no abnormal findings from interictal neurologic examination and various other test results.^25^

Meningoencephalitis of unknown origin was diagnosed if the dogs fulfilled the first 2 and at least 1 the remaining criteria among the following: the presence of (1) focal or multifocal central nervous system signs; (2) negative blood and CSF infectious disease titers (canine distemper, *Borrelia* spp., *Ehrlichia canis*, *Bartonella* spp., *Blastomyces* spp., *Cryptococcus* spp., *Neospora* spp., and *Toxoplasma* spp.); (3) MRI changes characterized by single or multiple T2 and fluid‐attenuated inversion recovery (FLAIR) hyperintense lesions within the brain; (4) CSF mononuclear pleocytosis; and (5) postmortem histopathological confirmation of MUO.

Brain tumor was diagnosed if the dogs fulfilled the first 3 and at least 1 the remaining criteria among the following^26^, ^27^: the presence of (1) at least 1 neurological symptom; (2) MRI changes characterized by solitary, regular‐shaped T2‐hyperintense, FLAIR‐hyperintense, and T1‐hypo/isointense lesions; (3) negative blood and CSF infectious disease titers; (4) postcontrast T1‐hyperintense lesions; (5) dural tail or dural contact; (6) mass effect; and (7) postmortem histopathological confirmation of BT.

### 2.2 Grouping

The epilepsy group consisted of IE dogs and dogs with BT or MUO showing seizures as 1 of the main signs of cerebral disease. The epilepsy group was divided into the IE group, which consisted of IE dogs, and structural epilepsy (SE) group, which consisted of epileptic MUO and BT dogs. The nonepileptic brain disease group included dogs diagnosed with MUO or BT that did not develop seizure during the follow‐up period, including the first visit.

### 2.3 Treatment

Dogs were diagnosed at first visit and treated for each neurologic disease; each treatment was as follows. Dogs with IE were treated with only antiepileptic drugs including phenobarbital, potassium bromide (KBr), or zonisamide. Among dogs diagnosed with MUO and BT, MUO dogs received immunosuppression‐based treatment, and dogs with BT received chemotherapy. If epilepsy was involved, additional antiepileptic drugs including phenobarbital, KBr, zonisamide, levetiracetam, or a combination of those antiepileptic drugs were prescribed.

### 2.4 Data collection

Data on characteristics including age, sex, epilepsy course, seizure frequency, concurrent disease, and epilepsy control status were collected after enrollment. In dogs with structural brain diseases, brain lesion volume was additionally included.

To examine the differences in serum HMGB1 concentrations in epileptic dogs, the epilepsy group was divided into different subgroups according to epilepsy course, seizure frequency, and status of epilepsy control. Epilepsy course was defined as the time from the initial onset of epilepsy to the first visit. As described in a previous report, the epileptic dogs were divided into 2 subgroups depending on the epilepsy course: dogs with an epilepsy course of >3 months and dogs with an epilepsy course of ≤3 months; furthermore, they were divided into 2 subgroups depending on the seizure frequency: dogs with a seizure frequency of ≤3 times/month and dogs with a seizure frequency of >3 times/month.^18^

Well‐controlled status was defined as the occurrence of <1 seizure/month after the initiation of epilepsy treatment.^28^ Poorly controlled status was defined as the occurrence of >1 seizure/month.

### 2.5 Assay

Blood samples (3 mL) were obtained from the jugular venipuncture within the first hour after the first visit. Sera were separated from the blood samples by centrifugation at 3500*g* and 4°C for 10 minutes within 1 hour of sample collection. Serum samples were stored as 1.5 mL aliquots at −80°C until the assays. The second set of serum samples was obtained at the follow‐up visit after the initiation of neurologic disease treatment. All serum samples were obtained within 72 hours of a seizure event.

Serum HMGB1 concentration was estimated using canine‐specific enzyme‐link immunosorbent assay (ELISA) kit (Dog HMGB1 ELISA kit, CUSABIO, Wuhan, China) according to the manufacturer’s protocol, for which the intra‐ and inter‐assay variabilities were < 8% and 10%, respectively. The detection limit was 0.02 ng/mL. The optical density was determined at 450 nm using an automated microplate reader (ELx 808, BioTek Instruments Inc, Winooski, Vermont).

### 2.6 MRI and brain lesion volume estimation

All dogs underwent MRI (0.3T, Airis II, Hitachi, Tokyo, Japan) of the brain at the first visit. All MRI data sets contained precontrast T1‐ and T2‐weighted images in at least 2 planes, at least a single planar FLAIR sequence, and postcontrast T1‐weighted images in at least 2 planes. T2‐hyperintense, FLAIR‐hyperintense, and T1‐hypo or isointense areas were defined as brain lesions.

The volume of each lesion was determined using commercial image analysis software (OsiriX 7.0, Osirix Foundation, Genova, Switzerland). Cerebral lesions were defined from transverse T2‐weighted images, and volumes of cerebral lesions were calculated with a commercial image analysis software, which accounts for interslice gaps.

### 2.7 Statistical analyses

Data were analyzed using a commercial statistical software (Prism 6, Graphpad Software Inc, La Jolla, California). Data are expressed as median (25th‐75th percentile). Two‐tailed test was used to calculate *P* values. The Kolmogorov‐Smirnov test was performed to determine whether data were normally distributed, and the following analyses were conducted using nonparametric tests because many subgroups did not follow a normal distribution. The Mann‐Whitney *U* test was used to compare the differences between 2 groups. The receiver operator characteristics (ROC) area under the curve was assessed to determine the optimal cutoff value of serum HMGB1 concentration for the differentiation between dogs with and without epilepsy. Spearman's test was used to analyze the correlation between sample interval and change in HMGB1 concentration (delta and ratio) and between brain lesion volume and HMGB1 concentration. The difference in serum HMGB1 concentration between the first visit and follow‐up visit (serum HMGB1 concentration at follow‐up visit—serum HMGB1 concentration at the first visit) was defined as delta, and the ratio of serum concentration at the first visit and follow‐up visit (serum HMGB1 concentration of follow‐up visit divided by serum HMGB1 concentration of first visit) was defined as ratio. *P*‐value <.05 was considered statistically significant.

## 3 RESULTS

### 3.1 Study population

The study group included 66 dogs, with 26 healthy control dogs and 40 dogs with brain diseases. Dogs with brain diseases included those with IE (14 dogs), epileptic MUO (10 dogs), epileptic BT (4 dogs), nonepileptic MUO (10 dogs), and nonepileptic BT (2 dogs). The median (±SD) serum HMGB1 concentration of healthy control dogs was 0.12 ± 0.34 (range, 0.02‐1.45) ng/mL.

### 3.2 Serum HMGB1 concentrations in dogs with seizures caused by different etiologies

**FIGURE 1:**
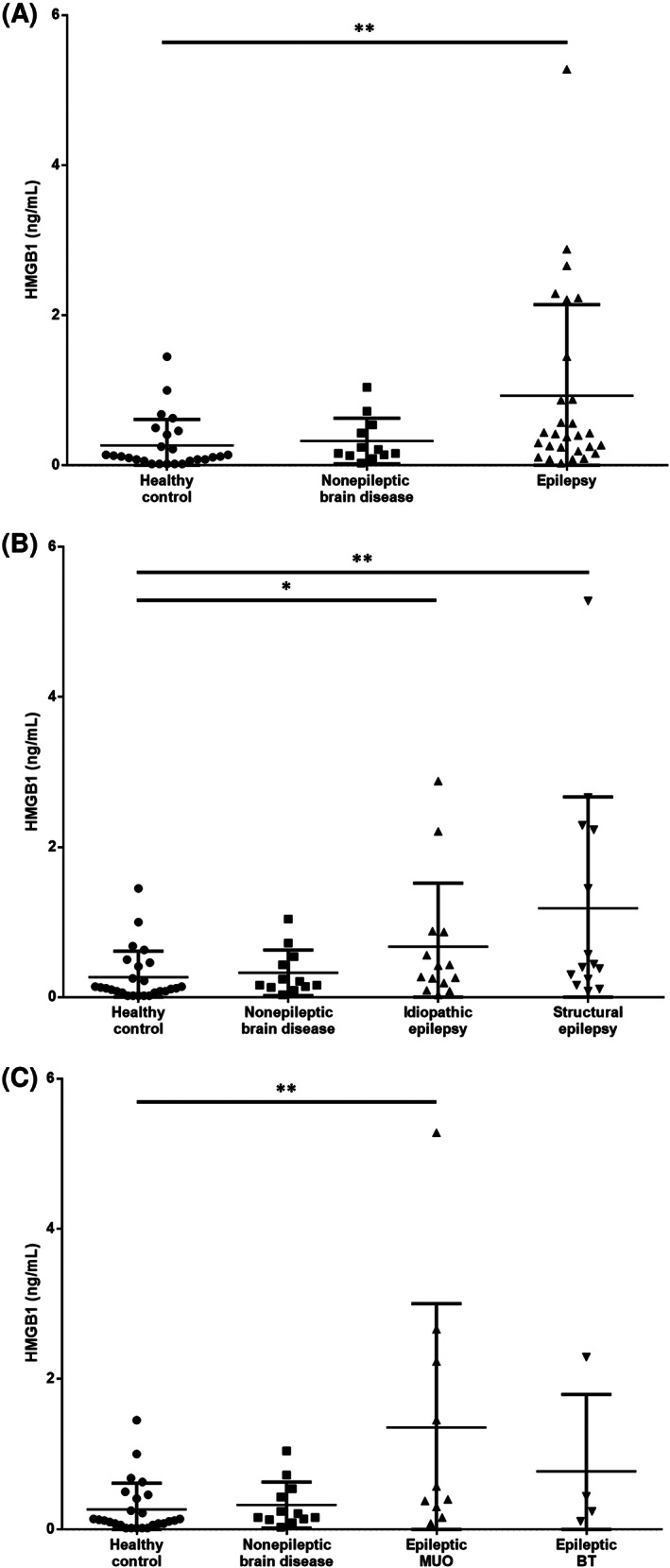
A, Serum HMGB1 concentrations of healthy control dogs compared to those of dogs with epilepsy and nonepileptic brain disease. B, Serum HMGB1 concentrations of healthy control dogs compared to those of dogs with idiopathic epilepsy, structural epilepsy, and nonepileptic brain disease. C, Serum HMGB1 concentrations of healthy control dogs compared to those of dogs with epileptic meningoencephalitis of unknown origin (epileptic MUO) and epileptic brain tumor (epileptic BT). The horizontal lines indicate the medians and interquartile ranges. The asterisk indicates statistically significant difference after Mann–Whitney *U* test (**P < .01, *P < .05)

The serum HMGB1 concentration of epileptic dogs was significantly higher than that of healthy control dogs (*P* = .002; Figure 1A). However, there were no significant differences in the concentrations of serum HMGB1 between healthy control dogs and dogs with non‐epileptic brain diseases (*P* = .12). With regard to seizure etiologies, serum HMGB1 concentrations of IE and SE dogs were significantly higher than those of the healthy control dogs (IE: *P* = .03, SE: *P* = .003; Figure 1B). However, there were no significant differences between the serum HMGB1 concentrations of IE and SE dogs (*P* = .41). In addition, unlike epileptic BT dogs (*P* = .16), epileptic MUO dogs showed a significantly higher serum HMGB1 concentration than did healthy control dogs (*P* = .005; Figure 1C).

### 3.3 ROC curve of serum HMGB1 concentration in canine epilepsy

**FIGURE 2:**
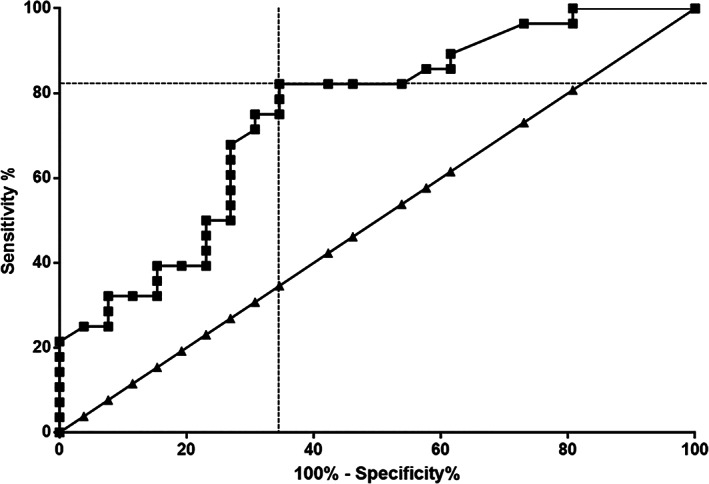
Receiver operating characteristic curve illustrating the sensitivity and specificity for the use of HMGB1 to distinguish dogs with epilepsy from those without epilepsy. The line marked by triangles represents the area under curve that is 50%. The area under the curve of the receiver operating characteristic curve line marked by rectangle symbols is 0.75 (95% confidence interval = 0.6134‐0.8769). The point of intersection indicates the optimal cutoff of 0.15 ng/mL for the differentiation between dogs with epilepsy and without epilepsy, with corresponding sensitivity and specificity of 82.1% (63.11%‐93.94%) and 65.4% (44.33%‐82.78%), respectively

The ROC area under the curve of epileptic dogs was 0.75 (95% confidence interval [CI] = 0.6134‐0.8769) (Figure 2). The cutoff value for serum HMGB1 concentration was determined as 0.15 ng/mL, as it exhibited the highest sensitivity (82.1%, 95% CI = 63.11%‐93.94%) and specificity (65.4%, 95% CI = 44.33%‐82.79%) for predicting canine epilepsy.

### 3.4 Comparison of serum HMGB1 concentrations among subgroups of epileptic dogs

With regard to the division of dogs according to epilepsy course, 18 and 9 epileptic dogs were included in the >3 months group and ≤ 3 months group, respectively (Table 1). No differences in serum HMGB1 concentrations were found between the >3 months and ≤3 months groups (*P* = .19). A similar result was observed in SE dogs (*P* = .77). However, among IE dogs, the >3 months group showed a significantly higher serum HMGB1 concentration than did the ≤3 months group (*P* = .02). Additionally, dogs with a seizure frequency of >3 times/month regardless of seizure etiologies presented insignificantly higher serum HMGB1 concentrations than did those with a seizure frequency of ≤3 times/month (epilepsy: *P* = .35, IE: *P* = .57, SE: *P* = .48).

**TABLE 1:** Comparison of serum HMGB1 concentrations among subgroups of epileptic dogs

	Epilepsy			IE			SE		
	Number (cases)	Median (ng/mL)	Range (ng/mL)	Number (cases)	Median (ng/mL)	Range (ng/mL)	Number (cases)	Median (ng/mL)	Range (ng/mL)
Epilepsy course									
≤ 3 month	18	0.325	0.03‐5.28	8	0.26	0.03‐0.88	10	0.505	0.08‐5.28
> 3 month	9	0.56	0.16‐2.88	5	0.87	0.42‐2.88	4	0.35	0.16‐2.66
*P*‐value		.19			.02*			.77	
Seizure frequency									
≤ 3 times/month	8	0.34	0.19‐0.43	5	0.27	0.19‐0.43	3	0.38	0.3‐0.4
> 3 times/month	20	0.57	0.03‐5.28	9	0.56	0.03‐2.88	11	1.01	0.08‐5.28
*P*‐value		.35			.57			.48	

Note: The asterisk indicates statistically significant difference after Mann–Whitney U test (*P < .05).

Abbreviations: IE, idiopathic epilepsy; SE, structural epilepsy.

### 3.5 Serum HMGB1 concentrations of epileptic dogs after treatment

Further investigations were carried out on 19 out of 28 epileptic dogs to identify changes in serum HMGB1 concentration after the treatment of underlying brain disease and epilepsy. In this analysis, 9 dogs with IE, 10 dogs with epileptic MUO, and 1 dog with epileptic BT that underwent treatment (sample interval) for 12 to 418 days (median 61 days) were included.

**FIGURE 3:**
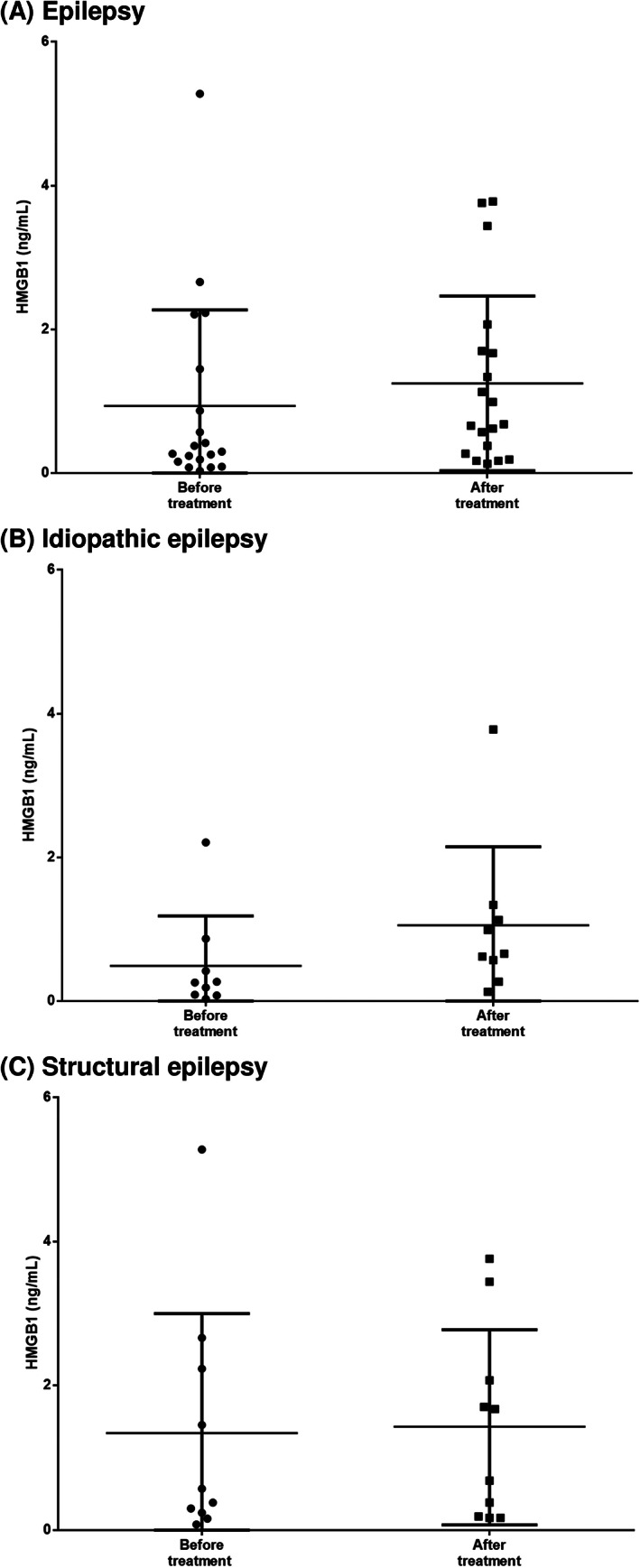
Comparison of serum HMGB1 concentration between before and after the treatment of epilepsy and underlying brain disease in epilepsy (*P* = .14; A), idiopathic epilepsy (*P* = .05; B), and structural epilepsy (*P* = .70; C)

All dogs with IE were treated with antiepileptic drugs, and all dogs with SE were treated with antiepileptic drugs and drugs for underlying brain diseases including MUO or BT. Serum HMGB1 concentrations of all these dogs after the initiation of epilepsy treatment regardless of the cause of epilepsy were higher than those before the initiation of epilepsy treatment (Figure 3). However, the level of elevation of serum HMGB1 in all groups was not significant.

### 3.6 Comparison of serum HMGB1 concentrations based on the status of epilepsy control

Status of epileptic dogs was classified into well‐controlled status and poorly controlled status according to the treatment response; 12 epileptic dogs showed a well‐controlled status, and 7 epileptic dogs showed poorly controlled status. There were no significant differences in serum HMGB1 concentrations before the initiation of the epilepsy treatment between dogs with well‐controlled and poorly controlled status. Furthermore, there were no significant differences regardless of epilepsy etiology (Table 2). Similar results were obtained after epilepsy treatment.

**TABLE 2:** Comparison of serum HMGB1 concentrations based on the status of epilepsy control

	Epilepsy			IE			SE		
	Number (cases)	Median (ng/mL)	Range (ng/mL)	Number (cases)	Median (ng/mL)	Range (ng/mL)	Number (cases)	Median (ng/mL)	Range (ng/mL)
Pretreatment									
Well‐controlled	12	0.34	0.08‐2.66	6	0.23	0.08‐2.21	6	0.92	0.24‐2.66
Poorly controlled	7	0.19	0.03‐3.44	3	0.26	0.03‐0.87	4	0.37	0.08‐5.28
*P*‐value		.35			.76			.56	
Posttreatment									
Well‐controlled	12	1.06	0.27‐3.78	6	0.83	0.27‐3.78	6	1.69	0.38‐3.76
Poorly controlled	7	0.19	0.13‐3.44	3	0.57	0.13‐1.34	4	0.18	0.17‐3.44
*P*‐value		.05			.48			.16	

Abbreviations: IE, idiopathic epilepsy; SE, structural epilepsy.

### 3.7 Correlation of serum HMGB1 concentration with the duration of treatment

**FIGURE 4:**
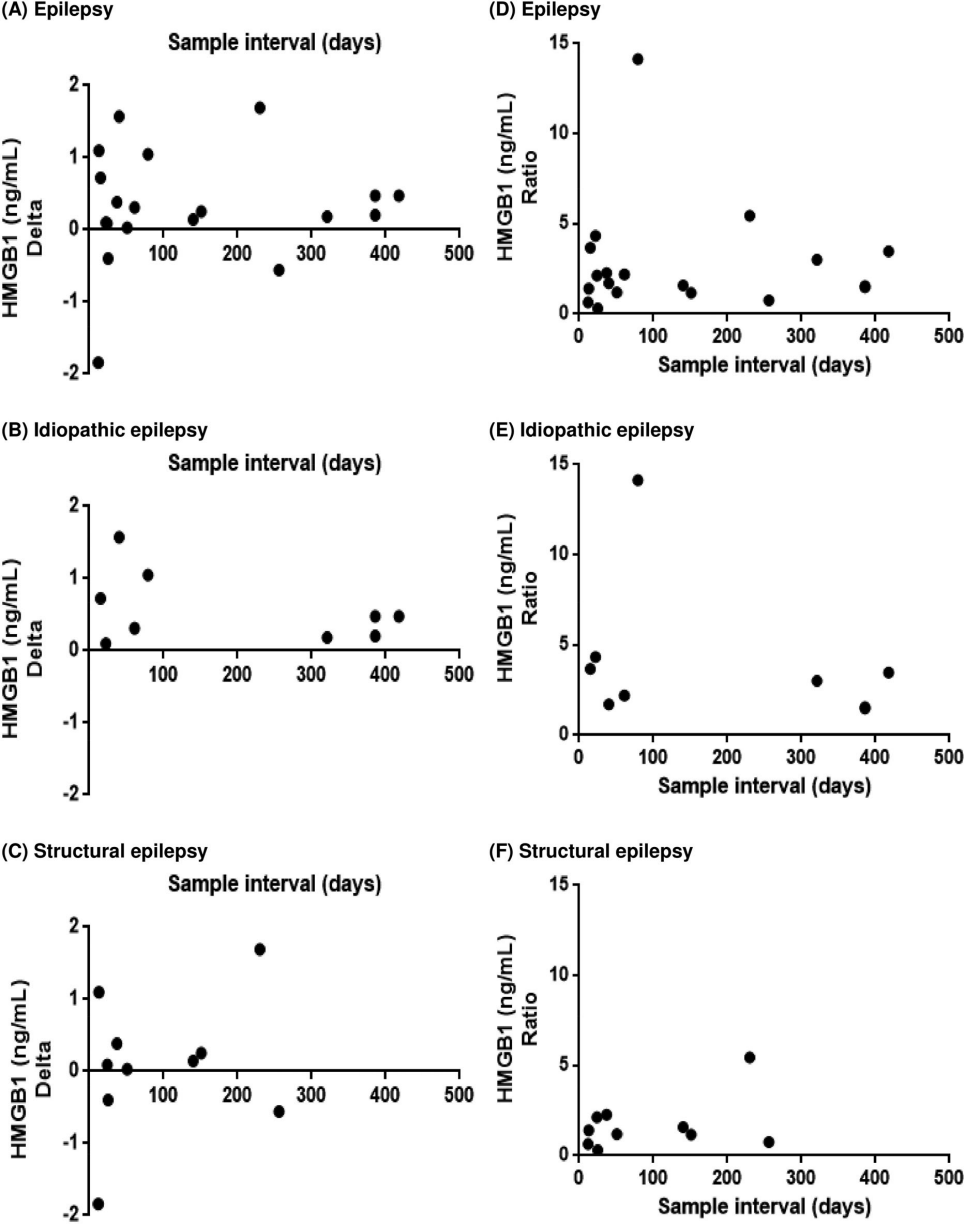
Correlation between delta and sample interval in dogs with epilepsy (*P* = .54, *r* = 0.15; A), idiopathic epilepsy (IE) (*P* = .72, *r* = −0.13; B), and structural epilepsy (SE) (*P* = .61, *r* = 0.19; C). Correlation between ratio and sample interval in dogs with epilepsy (*P* = .65, r = 0.11; D), IE (*P* = .24, *r* = −0.43; E), and SE (*P* = .61, *r* = 0.19; F)

To investigate the correlation between sample interval and serum HMGB1 concentration, which indicates the correlation between the duration of treatment and elevation of serum HMGB1 concentration, Spearman's correlation test was conducted. Both delta and ratio were found to be unrelated to the duration of treatment (Figure 4).

### 3.8 Correlation of serum HMGB1 concentration with brain lesion volume

**FIGURE 5:**
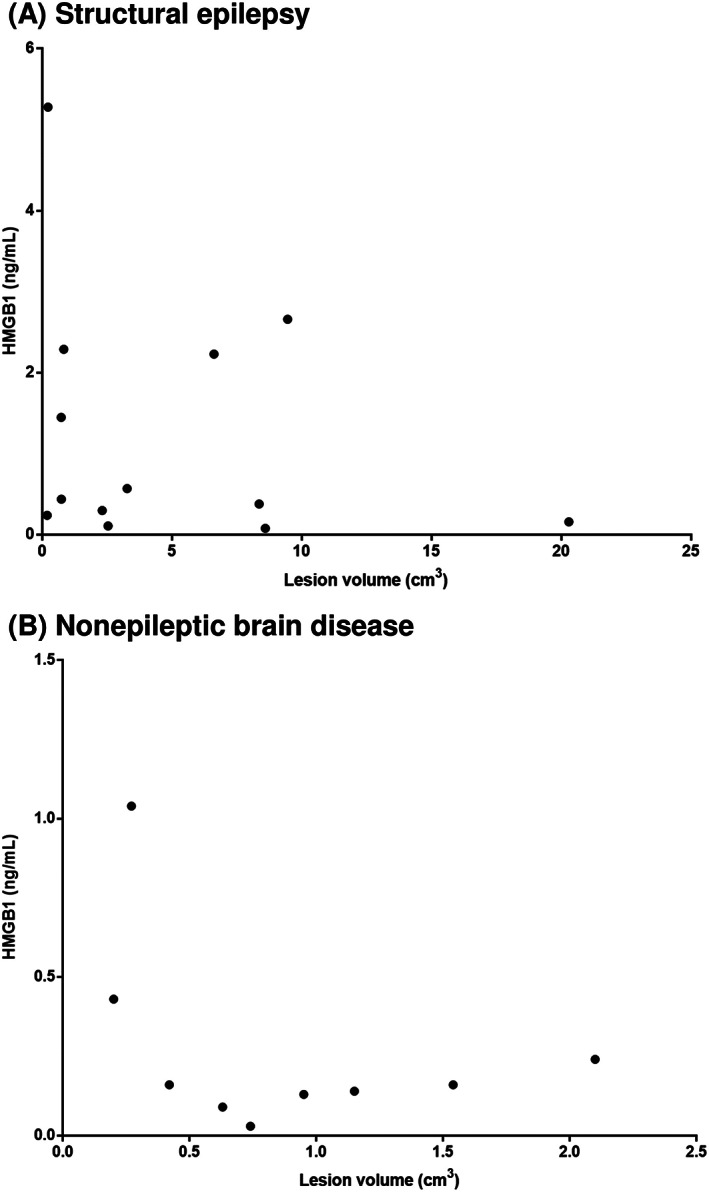
A, Correlation between serum HMGB1 concentrations and brain lesion volume (*P* = .42, *r* = −0.25) in dogs with structural epilepsy. B, Correlation between serum HMGB1 concentrations and brain lesion volume (*P* = .45, *r* = −0.2) in dogs with nonepileptic brain disease

Brain lesion volumes of dogs with SE were estimated to investigate the correlation between serum HMGB1 concentration and volume of brain lesion. Dogs with SE and those with nonepileptic brain diseases showed median (±SD) lesion volumes of 2.54 ± 5.52 (range, 0.19‐20.29) cm^3^ and 0.74 ± 0.58 (range, 0.2‐2.1) cm^3^, respectively. According to these results, there was no correlation between serum HMGB1 concentration and brain lesion volume in both SE dogs (*P* = .42, *r* = −0.247) and nonepileptic brain disease dogs (*P* = .45, *r* = −0.276) (Figure 5).

## 4 DISCUSSION

Serum HMGB1 concentrations were significantly higher in epileptic dogs than in healthy dogs, but not in those nonepileptic dogs with brain disease. In addition, serum HMGB1 concentrations of dogs with IE showing an epilepsy course of >3 months were also significantly higher than those of dogs with an epilepsy course of ≤3 months. However, other components including etiology of structural brain disease, seizure frequency, brain lesion volume, seizure control status, and duration of treatment were not related to serum HMGB1 concentrations in epileptic dogs. Taking our results into consideration, serum HMGB1 could be a biomarker of epilepsy or it might be associated with epileptogenesis in epileptic dogs, like in human patients with epilepsy.^15^, ^16^, ^17^, ^18^

It was recently confirmed that the expression of TLR‐4 signaling cascade including HMGB1 was higher in the brain tissue of dogs with epilepsy than in normal dogs.^14^ Cerebrospinal fluid reflects the dynamic changes caused by a central nervous system disease, but the process of obtaining a sample is more difficult and invasive than serum.^29^ However, recent report suggests that there is no correlation between CSF and serum concentrations of HMGB1 in patients with neurologic diseases.^29^ Furthermore, it has been demonstrated that the elevation of serum HMGB1 concentration is related to epilepsy in human patients.^18^ Therefore, considering the previously reported results and our results, which showed that serum HMGB1 concentration was increased in epileptic dogs, analysis of HMGB1 in serum might be more suitable than that in CSF in epileptic dogs. However, further studies on the measurement of HMGB1 concentration in CSF of epileptic dogs are needed to more clearly elucidate the correlation between HMGB1 and canine epilepsy.

Results of the present study are in line with a previous report about human patients with epilepsy showing higher serum HMGB1 concentration in the epilepsy group than in the healthy control group.^18^ Receiver operating characteristic curve analyses in the present study supported the assertion that serum HMGB1 concentration could be a biomarker for canine epilepsy. The cutoff value for serum HMGB1 concentration was determined as 0.15 ng/mL, as it exhibited the highest sensitivity (82.1%) and specificity (65.4%) for predicting canine epilepsy. Thus, serum HMGB1 concentration could be a useful screening tool for detecting canine epilepsy. To determine if there is a relationship between serum HMGB1 concentration and etiology of canine epilepsy, dogs with epilepsy were divided into those with IE and SE; serum HMGB1 concentrations of both IE and SE dogs were significantly higher than those of healthy control dogs. However, there was no difference between dogs with IE and SE. This result is consistent with the result of a previous study that compared IL‐1β levels in dogs with SE and IE.^13^ An increase in both HMGB1 and IL‐1β levels in epileptic dogs indicates that both inflammatory mediators are involved in the same inflammatory pathway, and it is assumed that the inflammatory pathway plays an important role in the development of canine epilepsy including structural and idiopathic etiologies. It has been reported that HMGB1 might play a role in the development of epileptogenesis by disruption of the BBB and induction neuroinflammation processes.^17^ Furthermore, regardless of the causes, increased HMGB1 expression and its secretion by glial cells during seizures is expected to support a second and sustained wave of extracellular HMGB1, which is distinct from the first wave caused by overexcited neurons at seizure onset.^30^ Both waves possibly enforce an autocrine and paracrine loop supporting neuronal hyperexcitability and sustaining inflammation via nuclear factor‐κB‐and/or activator protein‐1‐dependent transcription of inflammatory genes, including those of IL‐1β.^5^ Therefore, although it was not possible to be certain whether the increased serum HMGB1 level in the dogs was the cause or result of seizure, it could indicate the presence of epilepsy. Moreover, serum HMGB1 concentration was not significantly increased in dogs with nonepileptic brain diseases; however, serum HMGB1 concentration was significantly increased in epileptic dogs, suggesting that this inflammatory mediator is more related to epilepsy in itself than to structural brain diseases.

The previous report about factors, including seizure type, disease course, seizure duration and frequency, associated with the elevation of serum HMGB1 level in human patients with epilepsy concluded that seizure duration and frequency were more related with serum HMGB1 concentration.^18^ In the present study, the factors including seizure frequency and epilepsy course were investigated in epileptic, IE, and SE dogs, and the only significant result was found with regard to epilepsy course in IE dogs. Previous human studies have reported insignificant outcomes with regard to the disease course with a *P*‐value of .06, indicating a tendency. Thus, serum HMGB1 concentration might be associated with epileptic course in dogs with IE.

In the present study, serum HMGB1 concentrations in epileptic dogs, including those with structural and idiopathic causes, were measured before and after treatments. In both IE and SE dogs, serum HMGB1 concentrations were higher posttreatment than before the initiation of treatment. Previous reports have shown that as epilepsy treatment progressed, the levels of neuroinflammatory mediators decrease.^16^, ^31^, ^32^ The reason for the obtained result in the present study was presumed to be the difference in the commonly used antiepileptic drugs between humans and dogs. In human‐based epilepsy studies that reported a decrease in inflammatory mediators with the progression of epilepsy treatment, valproic acid was mainly administered.^16^, ^32^ Anti‐inflammatory effect of valproic acid has been reported; however, there are no reports of the anti‐inflammatory effects of phenobarbital and KBr, which are commonly administered to epileptic dogs.^33^ Therefore, the difference in the routinely prescribed antiepileptic drugs might have led to the differences in the effects of antiepileptic drugs on the serum HMGB1 concentrations in dogs and humans with epilepsy. Although the disease progression itself could increase serum HMGB1 concentrations in epileptic dogs after treatment, in the author's opinion, the possibility might be reduced considering that serum HMGB1 concentration was not related to brain lesion volume in dogs with structural epileptic diseases. Further longitudinal studies will be necessary to investigate the association between disease progression and serum HMGB1 concentrations in epileptic dogs.

Additional investigation was performed to determine the relationship between the increase in serum HMGB1 concentration and the duration of treatment. The increase in HMGB1 level was calculated using delta and ratio to investigate the correlation with the duration of treatment. However, the result showed that an increase in serum HMGB1 level is not correlated with treatment duration. Although there have been no reports on changes in serum HMGB1 concentrations with the administration of antiepileptic drugs, it has been reported that seizure frequency is associated with elevated serum HMGB1 concentration at the initial visit in human patients with epilepsy.^18^ Therefore, it was expected that there would be a change in serum HMGB1 concentration during epilepsy treatment according to the change in seizure frequency. However, according to the present study, there were no significant differences in serum HMGB1 concentrations between dogs showing well‐controlled status with low seizure frequency and those showing poorly controlled status with high seizure frequency. This result is consistent with the result that seizure frequency is not related to the elevation of serum HMGB1 concentration at the initial visit in the present study. Therefore, unlike the previous report about the relationship between human epilepsy and serum HMGB1 concentration,^18^ serum HMGB1 concentration and seizure frequency may not be related in dogs with epilepsy. However, this result could be because of the small number of cases. Therefore, further investigation consisting of large numbers of cases is needed.

One recent study demonstrated that serum HMGB1 concentration increases immediately after a generalized convulsive seizure and remains elevated for 6 hours.^34^ The times for each acquired serum sample after seizure varied in this study and thus, it is possible that variations in collection timing influenced the analysis of the correlation between seizure frequency and serum HMGB1 level. Therefore, future studies regarding changes in HMGB1 levels over time after seizure events in epileptic dogs are needed.

It has been reported that IL‐1β levels increase proportionately with the volume of brain lesions in patients with multiple sclerosis.^35^ In the present study, correlation between brain lesion volume and serum HMGB1 concentration was investigated in structural brain diseases in which inflammation occurs in the brain. There was no correlation between brain lesion volume and serum HMGB1 concentration, in dogs with or without epilepsy. Therefore, serum HMGB1 concentration could be interpreted to increase with the presence of epilepsy regardless of brain lesion volume.

This preliminary study had several limitations. First, the small number of dogs studied may have contributed to negative findings (Type 2 error). However, positive findings such as the elevation of serum HMGB1 concentrations in epileptic dogs could be attractive for the future researchers and be used to perform a sample size calculation for a larger cohort study. Second, the follow‐up recheck points were not coincident with all epileptic dogs. The differences in follow‐up recheck points could have led to unpredictable results, especially in the elevation of serum HMGB1 concentrations after the administration antiepileptic drugs. Therefore, the readers should interpret our results cautiously. A future study will be performed with more conservative follow‐up points.

In conclusion, increased serum concentration of HMGB1 was observed in dogs with both idiopathic and SE. However, serum HMGB1 concentration of dogs with nonepileptic brain disease was not significantly increased, indicating that serum HMGB1 could be a biomarker of canine epilepsy. Furthermore, the elevation of serum HMGB1 level in epileptic dogs was found to be independent of seizure frequency, seizure control status, duration of treatment, and brain lesion volume. However, it was found that the epilepsy course may be associated with elevation of serum HMGB1 level in dogs with IE. These results suggest that serum HMGB1 level could be used as a diagnostic biomarker for canine epilepsy.

## CONFLICT OF INTEREST DECLARATION

Authors declare no conflict of interest.

## OFF‐LABEL ANTIMICROBIAL DECLARATION

Authors declare no off‐label use of antimicrobials.

## INSTITUTIONAL ANIMAL CARE AND USE COMMITTEE (IACUC) OR OTHER APPROVAL DECLARATION

Authors declare no IACUC or other approval was needed.

## HUMAN ETHICS APPROVAL DECLARATION

Authors declare human ethics approval was not needed for this study.
